# Subcutaneous Uptake on [18F]Florbetaben PET/CT: a Case Report of Possible Amyloid-Beta Immune-Reactivity After COVID-19 Vaccination

**DOI:** 10.1007/s42399-021-01058-0

**Published:** 2021-09-14

**Authors:** Riccardo Laudicella, Irene Andrea Burger, Francesco Panasiti, Costanza Longo, Salvatore Scalisi, Fabio Minutoli, Sergio Baldari, Luigi Maria Edoardo Grimaldi, Pierpaolo Alongi

**Affiliations:** 1 Nuclear Medicine Unit, Fondazione Istituto G. Giglio, Contrada Pietrapollastra Pisciotto, Cefalù (Palermo), Italy; 2 Department of Biomedical and Dental Sciences and of Morpho-Functional Imaging, Nuclear Medicine Unit, University of Messina, Messina, Italy; 3 Department of Nuclear Medicine, University Hospital Zurich, University of Zurich, Zurich, Switzerland; 4 Department of Nuclear Medicine, Cantonal Hospital of Baden, Baden, Switzerland; 5 Neurology Unit, Fondazione Istituto G. Giglio, Contrada Pietrapollastra Pisciotto, Cefalù (Palermo), Italy

**Keywords:** COVID-19, Amyloid, PET/CT, Alzheimer, Florbetaben, Vaccination

## Abstract

**Introduction:**

Large-scale worldwide COVID-19 vaccination programs are being rapidly deployed, and high-risk patients with comorbidity are now receiving the first doses of the vaccine. Physicians should be, therefore, aware of new pitfalls associated with the current pandemic vaccination program, also in the case of [^18^F]Florbetaben PET/CT.

Case Presentation

We described the first image of [^18^F]Florbetaben PET/CT in the evaluation of a 70-year-old male with suspicious Alzheimer disease and unclear history of heart disease. We detailed the diagnostic imaging PET/CT workup with different findings.

**Conclusion:**

In this case, [^18^F]Florbetaben PET/CT can demonstrate potential beta-amyloid immune-reactivity and deposition associated with the current COVID-19 pandemic vaccination programs.

## Introduction

Large-scale worldwide COVID-19 vaccination programs are being rapidly deployed, and high-risk patients with comorbidity are now receiving the first doses of the vaccine. Physicians should be, therefore, aware of new pitfalls associated with the current pandemic vaccination program, also in the case of [^18^F]Florbetaben PET/CT.

## Case Presentation

**Fig. 1:**
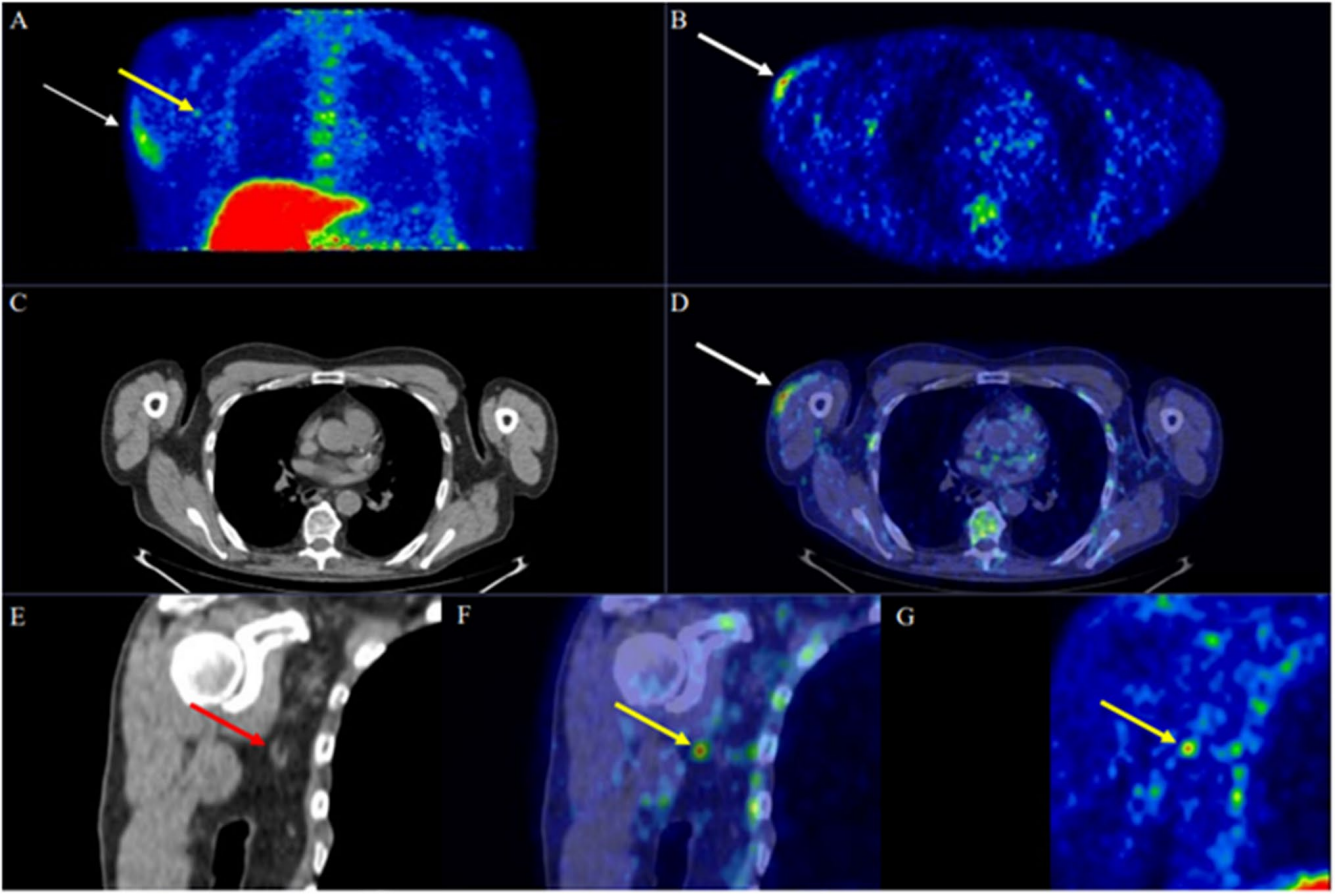
[^18^F]Florbetaben PET/CT: MIP (**A**), PET (axial-**B**, coronal-**G**), CT (axial-**C**, coronal-**E**), PET/CT (axial-**D**, coronal-**F**) images demonstrated ill-defined uptake in the right arm’s subcutaneous tissues (SUVmax 5.6; **white-arrows**) and next to a possible right-axillar lymph node (SUVmax 4.75; **yellow-arrows**) evident on low-dose CT scan without breathing control (**red arrows**). Reprinted with permission from Nuclear Medicine Unit, Fondazione Istituto G. Giglio, Cefalù (Palermo), Italy.

We report the case of a 70-year-old male who underwent [^18^F]Florbetaben PET/CT for suspected Alzheimer disease (AD) 1 day after the administration of the first dose of Pfizer-BioNTech COVID-19 vaccine in the right arm in absence of any related symptoms. The patient was also referred with a concomitant unclear history of heart disease (hypertensive disease and initial signs of heart failure with suspicious of cardiac amyloidosis), and therefore, the thorax was also included in the acquisition’s field due to [18F]Florbetaben PET potential utility in the diagnostic workup of cardiac amyloidosis [1, 2]. A moderate amyloid burden on the bilateral frontal and parietal brain cortex in the absence of cardiac beta-amyloid deposition was identified, referable to as the presence/development of AD. However, subcutaneous uptake on the vaccination site in the right arm’s deltoid region and focal uptake next to an ipsilateral axillary lymph node were noted. Tracer injection was via the left antecubital fossa, hence not a potential cause. In Fig. 1, [^18^F]Florbetaben MIP (A), PET (axial-B, coronal-G), CT (axial-C, coronal-E), PET/CT (axial-D, coronal-F) images demonstrated ill-defined uptake in the right arm’s subcutaneous tissues (SUVmax 5.6; white-arrows) and next to a possible right-axillar lymph node (SUVmax 4.75; yellow-arrows) evident on low-dose CT scan without breathing control (red arrows). We assume that the subcutaneous and the potential lymph node uptake might be related to an induced inflammation with peptides deposition, as amyloid-beta peptides are involved in the systemic inflammatory process such as in the physiopathology of AD, and chronic, low-level systemic inflammation may exacerbate the Aβ deposition [3–6]. Such induced inflammation, already demonstrated with several tracers [7–9], may be further enhanced in AD patients by the amyloid-beta presence. In this sense, Hsu et al. observed in a preclinical study that the intracellular immunoreactivity was significantly increased by the SARS-CoV-2 pseudovirus in the presence of Aβ_1-42_ (a strong indicator of AD with high affinity for SARS-CoV-2 spike protein S1 subunit) compared to SARS-CoV-2 pseudovirus alone. Interestingly, they also observed that Aβ_1-42_ significantly increased SARS-CoV-2 infectivity and that the clearance of Aβ_1-42_ can be reduced during SARS-CoV-2 infection [10]. Similar findings with other radiopharmaceuticals were recently described [7–9], but this is the first case to show that also [^18^F]Florbetaben PET/CT can demonstrate immune-induced findings, also amplified by the beta-amyloid presence, associated with the current COVID-19 pandemic vaccination programs, being a potential finding on whole-body protocols for the assessment of cardiac/systemic amyloidosis.

## Conclusion

[^18^F]Florbetaben PET/CT can demonstrate immune-induced findings, also amplified by the beta-amyloid presence, associated with the current COVID-19 pandemic vaccination programs.

## Data Availability

Data are available for bona fide researchers who request it from the authors.
